# Comparative Study on the Cellular and Systemic Nutrient Sensing and Intermediary Metabolism after Partial Replacement of Fishmeal by Meat and Bone Meal in the Diet of Turbot (*Scophthalmus maximus* L.)

**DOI:** 10.1371/journal.pone.0165708

**Published:** 2016-11-01

**Authors:** Fei Song, Dandan Xu, Kangsen Mai, Huihui Zhou, Wei Xu, Gen He

**Affiliations:** 1 The Key Laboratory of Aquaculture Nutrition and Feed (Ministry of Agriculture), Ocean University of China, Qingdao, 266003, China; 2 The Key Laboratory of Mariculture (Ministry of Education), Ocean University of China, Qingdao, 266003, China; Chinese Academy of Sciences, CHINA

## Abstract

This study was designed to examine the cellular and systemic nutrient sensing mechanisms as well as the intermediary metabolism responses in turbot (*Scophthalmus maximus* L.) fed with fishmeal diet (FM diet), 45% of FM replaced by meat and bone meal diet (MBM diet) or MBM diet supplemented with essential amino acids to match the amino acid profile of FM diet (MBM+AA diet). During the one month feeding trial, feed intake was not affected by the different diets. However, MBM diet caused significant reduction of specific growth rate and nutrient retentions. Compared with the FM diet, MBM diet down-regulated target of rapamycin (TOR) and insulin-like growth factor (IGFs) signaling pathways, whereas up-regulated the amino acid response (AAR) signaling pathway. Moreover, MBM diet significantly decreased glucose and lipid anabolism, while increased muscle protein degradation and lipid catabolism in liver. MBM+AA diet had no effects on improvement of MBM diet deficiencies. Compared with fasted, re-feeding markedly activated the TOR signaling pathway, IGF signaling pathway and glucose, lipid metabolism, while significantly depressed the protein degradation signaling pathway. These results thus provided a comprehensive display of molecular responses and a better explanation of deficiencies generated after fishmeal replacement by other protein sources.

## Introduction

Feeding-induced stimulation of anabolism not only depends on increased substrates for metabolic synthesis, but also requires activation of nutrient-sensing signaling pathways [1].Nutritional status is signaled at both cellar and systemic level [2]. The main mediator of cellular nutrient sensing is target of rapamycin (TOR) signaling pathway, which controls cell proliferation, protein synthesis [3] and intermediary metabolism [4, 5], while limitations of one or more essential amino acids (EAA) could activate the amino acid response (AAR) signaling pathway and results in general protein translational depression [6]. The coordination of TOR and AAR signaling pathway regulate cell protein synthesis and intermediary metabolism in response to altered nutritional status [7]. The insulin/insulin-like growth factor (IGF) system acts as the main systemic nutrient sensing pathway [8]. Binding of IGFs to its receptors regulates body growth through phosphoinositide-3-kinase (PI3K)/protein kinase B (AKT) pathway, which integrates with TOR pathways at multilevel to modulate tissue growth [9].

Recent findings have demonstrated that both TOR and IGF signaling pathways regulate intermediary metabolism [10, 11]. Activated TOR promotes translations of many enzymes in glucose, lipid and energy metabolism [12]. Similarly, suppressed IGF system via Forkhead-box Class O (FOXO) activates muscle protein ubiquitination through elevating the expression of E3-ubiquitin ligases Atrogin-1 and Muscle RING-finger 1 (Murf-1) [13]. These two regulators play a critical up-regulatory role in the initiation of ubiquitination [14]. Moreover, BCKDH-E2 is considered as the key enzyme in branch-chain amino acid (BCAA) catabolism which could accelerate the protein degradation process [15]. Study performed in zebrafish showed that high protein-low carbohydrate diet activates TOR signaling pathway and influenced the liver and muscle metabolism- and growth-related factors including glycolysis, lipogenesis myogenesis and muscle protein ubiquitination [16]. Furthermore, Azizi *et al*. [17] demonstrated that, lysine and leucine deficiencies down-regulated IGF system and TOR signaling, and decreased myogenesis in gilthead sea bream. Study on Fine Flounder demonstrated that fasting resulted in low production of muscle-derived IGF-I, and then through Akt/FoxO signaling increased the expression of MuRF-1and Atrogin-1[14].

Searching for alternative protein sources to replace fishmeal (FM) in aquafeeds represents a major challenge for aquaculture nutrition studies [18, 19]. Plant proteins and poultry by-products have been tested for their appropriate inclusion level in diets for a variety of aquaculture species [20–22]. The phenotypic responses, including growth performance and body composition have been characterized extensively. However, the underlying molecular responses after FM replacement that led to these phenotypes remain largely unexplored. We have recently reported that partial FM replacement by soybean meal in turbot led to postprandial hypo-activated TOR signaling and hyper-activated AAR signaling, which in turn suppressed postprandial anabolism [23]. To test whether these molecular responses are soybean meal specific or represented common reactions after FM replacement, we chose an animal protein, meat and bone meal (MBM), and examined the postprandial responses of turbot after MBM inclusion in its diet. In present study we not only focus on TOR and AAR pathways, but also pay close attention to the endocrine changes and intermediary metabolism regulations after FM replacement by MBM.

## Materials and Methods

### Animal ethics statement

All procedures performed in this study were in strict accordance with the Standard Operation Procedures (SOPs) of the Guide for the Use of Experimental Animals of Ocean University of China. Fish rearing procedures and sampling were approved by the Institutional Animal Care and Use Committee of Ocean University of China(Permit Number: 20001001).All the fish were anesthetized with benzocaine (30mg/L) to minimize suffering before being assigned to the tanks, weighed and sampling, and all efforts were made to minimize suffering.

### Diet formulation and fish rearing

Three isonitrogenous (50.1% crude protein) and isoenergetic (20.8 KJ/g) practical diets were formulated to contain different protein sources: 60% FM (FM diet, control group); 45% FM protein replaced by MBM containing33% FM + 34.6% MBM (MBM diet) and MBM diet supplemented essential amino acids (EAAs) mixture to match the EAA profile of FM diet (MBM+AA diet). All ingredients were first ground into fine powder through a 320μm sieve before diet preparation. The ingredients were blended thoroughly and extruded as pellets. All the experimental diets were dried for 12 h at 45°C and packed in plastic bags and refrigerated at -20°C until used. The formulation and composition of the three experimental diets were listed in Table 1 and their amino acid profiles were provided in Table 2.

**Table 1:** Composition of the experimental diets.

Ingredients (g/100g)	Diets (Dry weight)		
	FM	MBM	MBM+AA
Fishmeal ^a^	60	33	33
Meat and bone meal ^b^	0	34.2	34.2
Wheat gluten meal ^c^	3	5	3.81
Wheat meal	22.6	13.4	13.4
Beer yeast	2	2	2
Amino acid mixture ^d^	0	0	1.19
Fish oil	3	5	5
Palm oil	1.5	0	0
Lecithin	2.5	2.5	2.5
Mineral premix ^e^	1.5	1.5	1.5
Vitamin premix ^f^	1.5	1.5	1.5
Choline chloride	0.25	0.25	0.25
Attractant ^g^	1	1	1
Mold inhibitor	0.1	0.1	0.1
Antioxidant	0.05	0.05	0.05
Taurine	0	0.5	0.5
MCC	1	0	0
Total	100	100	100
**Analytical composition (dry matter basis) g/100g**			
Dry matter	94.97	94.72	94.76
Crude protein	50.14	50.15	50.1
Crude lipid	11.61	11.82	11.8
Gross energy (KJ/g)	20.7	20	19.8

FM, fishmeal; MBM, meat and bone meal replacement diet; MBM+AA, MBM diet with dietary essential amino acid supplementation.

^a^ Fishmeal: steam dried fishmeal, with crude protein: 70.10%, crude lipid: 7.58%,supplied by COPEINCA Group (Lima, Peru).

^b^ Meat and bone meal: Meat and bone meal, with crude protein: 55.34%,crude lipid: 10.04%, supplied by Australia.

^c^ Wheat gluten meal and wheat meal act as carbohydrate source and filler, supplied by Great Seven Bio-tech. Co., Ltd (Shandong, China).

^d^ Amino acid mixture(g/kg diet): L-Methionine (Coated amino acid obtained, 90%),2.4; L-Lysine (Coated amino acid, 60%), 6.1; L-Leucine (Crystalline amino acid, 99.4), 1.6; L-Histidine (Crystalline amino acid, 99.1%),0.5; L-Threonine (Crystalline amino acid, 99.9%), 1.3. Coated amino acid supplied by Beijing XingHuo Yuan Science and Technology Co., Ltd.Crystalline amino acid supplied byJizhou City Huayang Chemical Co., Ltd.

^e^ Mineral premix (mg/kg diet): CoCl_2_·6H_2_O (1%), 50; CuSO_4_·5H_2_O (25%), 10; FeSO_4_·H_2_O (30%), 80; ZnSO_4_·H_2_O (34.50%), 50; MnSO_4_·H_2_O(31.80%), 45; MgSO_4_·7H_2_O(15%), 1200; Sodium selenite (1%), 20; Calcium iodine (1%) 60; Zeolite, 11470.

^f^ Vitamin premix(mg/kg diet): thiamin (98%), 25; riboflavin (80%), 45; pyridoxine-HCl (99%), 20; vitamin B_12_ (1%), 10; vitamin K_3_ (51%), 10; inositol (98%), 800; pantothenic acid (98%), 60; niacin acid (99%), 200; folic acid (98%), 20; biotin (2%), 60; retinol acetate (500000 IU/g), 32; cholecalciferol (500000 IU/g), 5; alpha-tocopherol (50%), 240; ascorbic acid (35%), 2000; anti-oxidants (oxygen ling grams, 100%), 3; rice husk powder (100%), 11470.

^g^ Attractant: (betaine: dimethylpro-piothetin: glycine: alanine: inosine5'-phosphate =

4:2:2:1:1).

**Table 2:** Amino acids composition of the diets (%).

Amino acid	FM	MBM	MBM+AA
Essential amino acids			
Leucine	3.47	3.31	3.43
Methionine	1.26	1.06	1.20
Lysine	3.43	2.98	3.38
Threonine	2.10	1.77	1.98
Arginine	2.99	3.02	3.04
Histidine	1.12	1.04	1.16
Phenylalanine	2.11	2.17	2.18
Isoleucine	1.63	1.78	1.74
Valine	2.18	1.99	2.11
Total EAA ^a^	20.29	19.12	20.22
**Non-essential amino acids**			
Glycine	3.28	4.17	4.45
Aspartic acid	4.26	3.62	3.83
Serine	2.51	2.23	2.34
Proline	2.50	3.17	3.23
Cysteine	1.13	1.17	1.09
Tyrosine	1.38	1.38	1.15
Alanine	2.98	3.06	3.22
Glutamic acid	7.84	7.29	7.39
Total NEAA ^b^	25.88	26.09	26.71
EAA/NEAA	0.78	0.73	0.76

FM, fishmeal; MBM, meat and bone meal replacement diet; MBM+AA, MBM diet with dietary essential amino acid supplementation.

^a^ ΣEAA: sum of essential amino acid

^b^ ΣNEAA: sum of non-essential amino acid

Juvenile turbots were purchased from Haiyang fish farm (Shandong, China). Before the feeding trial, juvenile turbots were reared in tanks filled with 500 liters of seawater and were fed a commercial diet supplied by Great Seven Bio-tech. Co., Ltd(Shandong, China) twice per day for two weeks to acclimate to the experimental conditions. At the beginning of the experiment, turbot were fasted 24h and weighed in order to obtain the initial body weight. Mixed-sex turbots with an initial weight of 9.19±0.01g were evenly and randomly assigned into tanks filled with 500 liters of seawater, with 40 fish per tank. Each diet was randomly allocated in triplicate tanks. Fish were hand-fed twice daily (07:00 and 17:00) to apparent satiation for one month. During the one month feeding trail, the water temperature ranged from 19 to 22°C, dissolved oxygen ranged from 6.0 to 7.0 mg/L and salinity ranged from 29 to 33‰.

### Sample collection

Before the experiment, twenty turbots were randomly selected in order to measure the initial whole body compositions. Following the one month feeding trial, fish were fasted for 48h to get the basal level of body metabolism [24, 25]. After being fasted for 24h, all experimental fish from each tank were weighed and counted, and four fish from each tank were randomly collected and stored at -20°C for finial biochemical analysis. At 48 h after the last feeding, sampled fish at the end of fasting period were designated as 0h samples. Six fish (two per tank) from every treatment (n = 6) were collected at 2, 8 and 24 h after re-feeding. After anaesthetised with benzocaine (30mg/L), fish were killed by cervical section. Blood samples were taken from the caudal vein using 1mL heparin anti-coagulation syringe to obtain plasma samples and centrifuged at 3,000 g for 5 min at 4°C. The plasma was removed into a new tube and immediately transferred to liquid nitrogen and then kept at -80°C until analysis. Liver and dorso-lateral white muscle from six fish in each treatment were excised and pooled into 1.5 ml tubes (RNAase-Free; Axygen), frozen in liquid N_2_ and then stored at -80°C for later analysis.

### Biochemical analyses

Whole body moisture content was analyzed by drying the samples to constant weight at 105°C. Crude protein of experimental diets and fish samples was determined by digestion using the Kjeldahl method (Kjeltec TM 8400, FOSS, Sweden). Crude lipid was measured by ether extraction using the Soxhlet method (Buchi 36680, Switzerland). An adiabatic bomb calorimeter was used to measure the gross energy of feed and fish samples (C2000, IkaWerke). Ingredients and diets were dried by the freeze dryer (ALPHA1-2 LDplus, Christ co., Ltd, Germany) and then measured by L-8900 amino acid analyzer (Hitachi, Japan). The Plasma triglycerides (TG) and glucose (GLU) concentrations were analyzed using commercial kits according to the instructions (Sysmex) [23, 26].

### Quantification of gene expression by real-time PCR

To examine level of gene expression, real-time quantitative PCR (qRT-PCR) were carried out in a quantitative thermal cycle (Mastercycler, Eppendrof, Germany). The prime sequences for all the target genes were designed based on each sequences (Table 3). Total RNA was isolated from muscle and liver samples using Trizol Reagent (Invitrogen, USA). Isolated RNA quantity and quality were determined via spectrophotometry using a NanoDrop 2000 spectrophotometer and on a 1.2% denaturing agarose gel, respectively. Total RNA (1μg) was reverse transcribed into cDNA by PrimeScript^TM^ RT reagent Kit (Takara, Japan) according to the instructions and cDNA was diluted into 80 ng/μL by RNase-free water. The amplification was performed in a total volume of 25μL, containing 12.5μL of 2×SYBR Premix Ex Taq^TM^ II (Takara, Japan), 2μL of the cDNA product, 0.5μL of each primer (10 mM) and 9.5μL of Rnase-free water. The qRT-PCR conditions were: 95°C for 2min, followed by forty cycles consisting of 95°C for 10 s, 58–60°C for 10 s, and 72°C for 20 s. When PCR amplification was finished, melting curve analysis was performed to verify that a single amplified product was present. The qRT-PCR analyses focused on the key regulators of IGF system, which were as follow: insulin-like growth-factor-I (IGF-I) and insulin-like growth-factor-II (IGF-II); insulin-like growth-factor I receptor (IGF-IR), insulin-like growth-factor binding protein 1 (IGFBP-1), insulin-like growth-factor binding protein 2 (IGFBP-2), insulin-like growth-factor binding protein 4 (IGFBP-4), insulin-like growth-factor binding protein 5 (IGFBP-5), insulin-like growth-factor binding protein 6 (IGFBP-6) mediating IGFs biologic actions [16]. Furthermore, qRT-PCR also followed the transcript level of key enzymes and regulators involved in protein ubiquitination and hepatic intermediary metabolism: Atrogin-1 and muscle RING-finger 1 (Murf-1), key transcription factors of muscle ubiquitination; branched chain alpha keto acid dehydrogenase E2 subunit (BCKDH-E2) for branched-chain amino acids catabolism; pyruvate kinase (PK) and glucokinase (GK) for glycolysis; glucose 6 phosphatase 1 (G6Pase1) and fructose-1,6-bisphosphatase (FBPase) for gluconeogenesis; fatty acid synthetase (FAS) and a relevant transcription factor sterol regulatory element-binding protein 1 (SREBP1) for fatty acid synthesis; diacylglycerol O-acyltransferase homolog 1 (DGAT1) and diacylglycerol O-acyltransferase homolog 2 (DGAT2) for triglyceride synthesis; acyl-CoA oxidase 1 (ACOX1) and carnitine palmitoryltransferase 1 isoform a (CPT-1a) for fatty acid oxidation [3, 5, 16]. RNA polymerase II subunit D (RPSD) and elongation factor 1α (EF1α) were used as the reference genes as they were stably expressed among treatments. The expression level of target genes were calculated by 2^-(△△Ct)^ method [27].The value of FM diet at 0h was considered as control value.

**Table 3:** The primer pairs sequences used for qRT-PCR.

Gene	Forward primer (5'-3')	Reverse primer (5'-3')	Amplicon size (bp)	Ta (°C)	Accession no.
IGF-I	GGTGGACGAGTGCTGCTTC	CTGCCCTGCGGTACTAACC	145	59	FJ160587.1
IGF-II	ACCAACTGGGAAACTAACTCA	CACAGCTACGGAAACAACAT	424	56	JN032705.1
IGF-IR	GATGTGCGGGCGTACTCGA	ATCCGGCTTGGTGGGCTTG	228	58	AJ224993.1
IGFBP-1	CGCAACAAACTGGTACAACAGG	GTCACCGGGCAGGTCACTC	246	59	JF441174.1
IGFBP-2	GGTCCAGATACCAGCGTCAC	CCGCAGACAAAGTAGGGAAG	241	58	HQ696115.1
IGFBP-4	CGCAGGGTGACACTGTTTA	ATCCTCCCAAATCCACGAA	200	59	**- - -**
IGFBP-5 ^a^	GAGTTCCAGCCAGCCAAAG	GCCATTACGCGAGAAGTGT	241	58	**- - -**
IGFBP-6 ^a^	GGCTCTGTTGCAGGGACG	GCGAGTGTCACAGTTGGGTAT	207	59	**- - -**
Murf-1 ^a^	CTGCCGCTTCGAGGTGAT	TGATGCGTTCGTCTTCGTG	185	58	**- - -**
Atrogin-1 ^a^	AGGAGAACTTGCTGCTGTCG	AGATCCAAGCGGTTGAAGG	184	58	**- - -**
BCKDH-E2 ^a^	CTGCTCAGCGTGTTGGATG	GAGGGAGAATCACTGGTTTGG	157	60	**- - -**
PK	TGGATACGCTGAAGGAGATG	ACGCACGTTCTTGATGGTC	236	60	DQ848903
GK	CGACACGAGGACATTGACAAG	CCAACAATCATCCCGACTTCAC	218	60	JX678944
G6Pase1	CACGAGACGGCTCATTATGC	CTTTGCTGCTGGATTTCTTGC	193	58	KC184131
FBPase	CAGGAAGGCTGGGATCGCTAAC	CTCATCTTCCTCCGACACAAG	157	60	KC184130
FAS	GGCAACAACACGGATGGATAC	CTCGCTTTGATTGACAGAACAC	195	58	KC189927
SREBP1 ^a^	GCCATTGACTACATCCGTTAC	CATCAGCCTGTCCATCTACTTC	136	60	**- - -**
DGAT1	ATACTCGTGTCCATCTGTGTCTC	AGTCGTCTCATCAGGAACCTTAC	177	58	KC189938
DGAT2	TGCTGTGGTCATCGTTATC	CTTGTAGGCGTCGTTCTC	163	56	KC189939
ACOX1	AGTCCTCGCCCAGCTTTACT	GGCTTCACATAGGTTCCGTCT	240	58	KC189925
CPT1A	ATGGGAAGAGTGGACTGAATG	GCTGGAAGGCATCTGTGG	96	58	KC189926
RPSD	CTGCTGTTCCCTAAAGAGTTCG	GAGCCGTGTAGTTCAGGGTCT	151	58–60	DQ848899.1
EF1α	TCATTGGCCATGTCGACTCC	ACGTAGTACTTGGCGGTCTC	226	56–60	AF467776.1

IGF-I, insulin-like growth-factor-I; IGF-II, insulin-like growth-factor-II; IGF-IR, insulin-like growth-factor I receptor; IGFBP-1, insulin-like growth-factor binding protein 1; IGFBP-2, insulin-like growth-factor binding protein 2; PK, pyruvate kinase; GK, glucokinase; G6Pase1, glucose 6 phosphatase 1; FBPase, fructose-1,6-bisphosphatase; FAS, fatty acid synthetase; DGAT1, diacylglycerol O-acyltransferase homolog 1; DGAT2, diacylglycerol O-acyltransferase homolog 2; ACOX1, acyl-CoA oxidase 1; CPT1A, carnitine palmitoryltransferase 1 isoform A; RPSD, RNA polymerase II subunit D; EF1α, elongation factor-1 alpha.

^a^ partial sequences of target genes in turbot were obtained through a degenerate PCR strategy in this study, including IGFBP-4, insulin-like growth-factor binding protein 4; IGFBP-5, insulin-like growth-factor binding protein 5; IGFBP-6, insulin-like growth-factor binding protein 6; Murf-1, muscle RING-finger 1, Atrogin-1; BCKDH-E2, branch-chain α-keto acid dehydrogenase E2 subunit and SREBP1, sterol regulatory element-binding protein 1.The partial sequence of those genes were presented in the S1 File.

### Western blot analysis

Relative protein level for phospho-TOR, TOR, phospho-AKT, AKT, phosphor-eIF2α, eIF2α, phospho-4E-BP1, 4E-BP1, phospho-ribosomal protein S6 (S6), S6, ATF4 and muscle RING-finger 1 (Murf-1) were analyzed by the western blotting. The frozen liver and muscle samples (50mg) were lysed in RIPA buffer containing 150mM NaCl, 50mM Tris, 0.5% NP-40, 1mM EDTA, 0.1% SDS, PH = 7.5), plus protease and phosphatase inhibitors cocktail (Roche, USA). Protein concentrations were measured by an Enhanced BCA Protein Assay Kit (Beyotime Biotechnology). Equal amounts of lysates (10 μg) were electrophoresed on sodium dodecyl sulfate-polyacrylamide gels (SDS-PAGE) and transferred to 0.45μm PVDF membrane (Millipore, USA) and blocked with 5% non-fat milk. Sample were incubated with the primary antibodies (4°C overnight) against β-tubulin, phospho-TOR, TOR, phospho-AKT, AKT, phosphor-eIF2α, eIF2α, phospho-S6, S6, phospho-4E-BP1, 4E-BP1 (Cell Signaling Technology, USA), ATF4 (Santa Cruz Biotechnology Inc, USA) and Murf-1 (Abcam Biotechnology Cambridge) and visualized with secondary antibody (HRP-labeled goat anti-Rabbit IgG) (Beyotime Biotechnology) at 1:10000 dilution for 1 h at the room temperature. The membrane was exposed to the X-ray film, and the band densities quantified by the NIH Image 1.63 software. All the antigenic region against the antibodies used in this study were confirmed to be conserved in turbot and successfully used in previous studies [23].

### Statistical analysis

All results data were expressed as means ± standard error of means. The software SPSS 17.0 was used for all statistical evaluations. The data of growth performance were subjected to one-way analysis of variance (ANOVA) followed by Tukey’s multiple range tests. A two-way ANOVA testing was used to determine the effects of diets (D), re-feeding time (T) and their interaction on the nutrient response. Data were transformed into logarithms, square roots or reciprocals to meet the ANOVA criteria when data were nonparametric or not homoscedastic. When significant interactions were found, the value of each treatment was compared with the control value by Shaprio-Wilk test [23]. Value of *P<0*.*05* were considered statistically significant.

## Results

### Phenotypic responses and feed intake

As shown in Table 4, there were no significant differences of feed intake and survival rate of turbot for all dietary groups (*P>0*.*05*) after 4 weeks cultural experiment. However, feed efficiency rate was significantly lower in the MBM and MBM+AA diet groups compared with the FM diet group (*P = 0*.*01*). Furthermore, in comparison to the FM diet group, the retention of body energy, fat and protein were deceased in MBM and in MBM+AA diet groups (*P≤0*.*01*).The specific growth rate, whole body protein and fat content of fish fed MBM and MBM+AA diet were significantly lower than that of fish in the control treatment group(*P<0*.*05*).

**Table 4:** Growth performance and nutrient utilization of turbot after one-month feeding trial on experimental diets.

	FM	MBM	MBM+AA	P-value
Initial body weight (g)	9.19±0.01	9.10±0.07	9.19±0.01	0.27
Final body weight (g)	28.79±1.01^a^	21.87±0.74^b^	22.96±0.34^b^	<0.001
Specific growth rate (%/d) ^a^	3.26±0.10^a^	2.50±0.08^b^	2.61±0.04^b^	<0.001
Feed efficiency rate ^b^	1.52±0.06^a^	1.27±0.04^b^	1.32±0.02^b^	0.01
Survival rate (%) ^c^	100.00	100.00	99.17±0.83	0.42
Feed intake (%/d) ^d^	2.27±0.13	2.16±0.06	2.16±0.03	0.59
Protein retention (%) ^e^	48.08±1.94^a^	38.32±1.08^b^	38.51±0.88^b^	<0.001
Fat retention (%) ^e^	53.83±2.30^a^	33.81±2.25^b^	35.03±2.38^b^	<0.001
Energy retention (%)^e^	36.59±1.45^a^	29.82±1.05^b^	29.32±1.20^b^	0.01
Whole body protein content (% w.w. ^f^)	15.40±0.14^a^	14.78±0.06^b^	14.48±0.11^b^	<0.001
Whole body fat content (% w.w. ^f^)	3.47±0.17^a^	2.83±0.09^b^	2.85±0.13^b^	0.02

FM, fishmeal; MBM, meat and bone meal replacement diet; MBM+AA, MBM diet with dietary essential amino acid supplementation.

Values are means ± SE, n = 3. Mean values in the same column with different superscripted small letters mean significant difference (P<0.05).

^a^ Specific growth rate (%/d) = 100×[ln (final body weight)−ln (initial body weight)]/d

^b^ Feed efficiency = wet weight gain (g)/total feed consumed (g).

^c^ Survival rate (%) = 100×(final fish number/initial fish number).

^d^ Feed intake (% body weight/d) = 100×feed consumption/(30 d × (initial body weight + final body weight)/2).

^e^ Nutrient retention = [100×(final body weight×final carcass nutrient content)−(initial body weight×initial carcass nutrient content)]/nutrient intake, where nutrient refers to protein, lipid and energy

^f^ w. w.: wet weight

### Cellular nutrient sensing pathways

**Fig 1:**
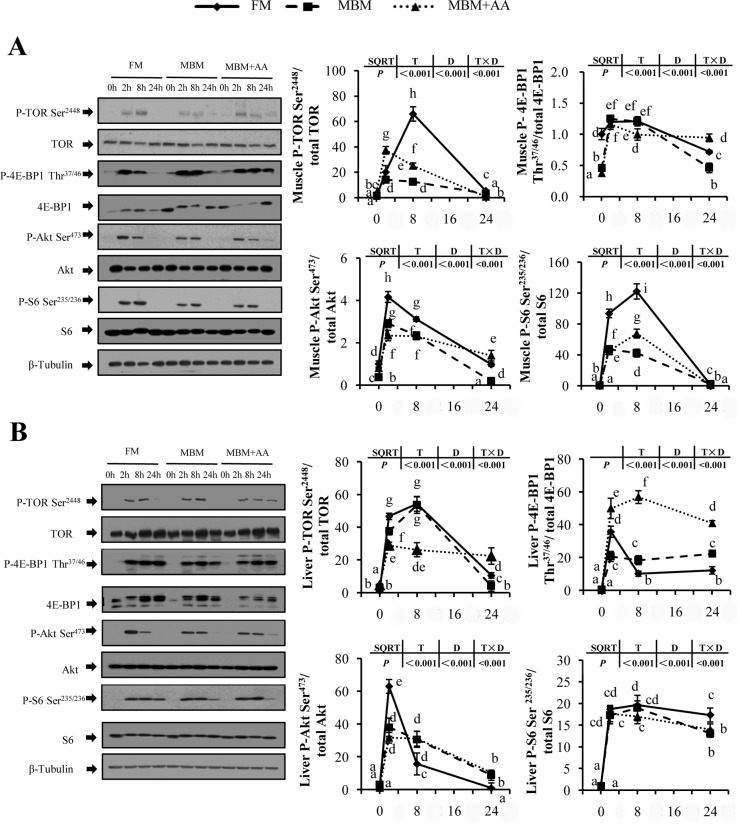
**Post-prandial expression profile of TOR signaling pathway in muscle (A) and liver (B) of juvenile turbot.** Values are means ± SE, n = 6. All results were analyzed using two-way ANOVA followed by Tukey’s test. Data are expressed relative to reference protein Tubulin. When the interaction was significant (*P < 0*.*05*), “a,b,c,d,e,f,g,h,i” mean different from all treatments. On the contrary, “A,B,C,D” were used to represented the significant difference from time points and “X,Y,Z” were used to represented the significant difference from diets (*P<0*.*05*). T, D and T×D represented time points, diets and interaction between T and D, respectively. SQRT indicates that data were transformed and statistically analyzed with square roots.

As shown in Fig 1, re-feeding markedly activated the TOR signaling pathway with high phosphorylation level of TOR, AKT, S6 and 4E-BP1 in both muscle and liver *(P<0*.*05)*. In muscle, the peak value of postprandial phosphorylated TOR increased approximately 64.9-fold, 13.1-fold and 36.3-fold after re-feeding with FM diet, MBM diet and MBM+AA diet, respectively compared to those of fasting samples *(P<0*.*05)*. Compared with the FM diet, MBM diet and MBM+AA diet markedly decreased the muscle and liver phosphorylation level of the key proteins associated with TOR signaling *(P<0*.*05)*. Specifically, compared with the FM diet, MBM diet and MBM+AA diet significantly decreased the phosphorylation level of S6 by about 3.0-fold, 2.0-fold at 8h after re-feeding each diet in muscle *(P<0*.*05)*.

**Fig 2:**
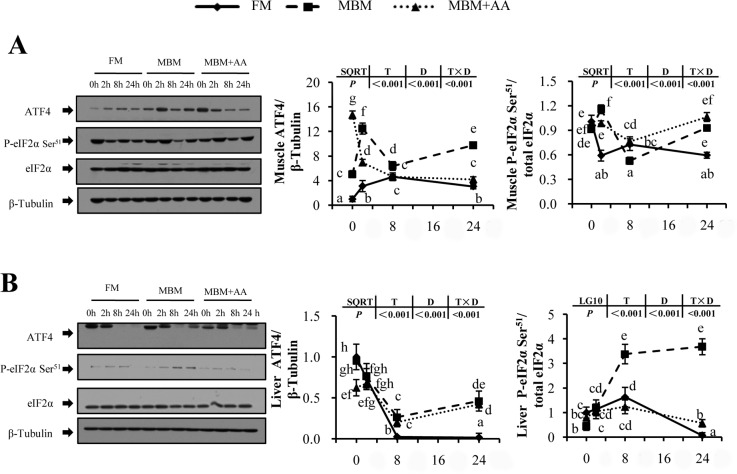
**Post-prandial expression profile of AAR signaling pathway in muscle (A) and liver (B) of juvenile turbot.** Results are represented as means with standard errors (n = 6) and were analyzed using two-way ANOVA followed by Tukey’s multiple range test. The different superscript letters (‘a,b,c,d,e,f,g,h’) mean significantly different among the values (*P<0*.*05*). LG10 indicates that data were transformed and statistically analyzed with log transforms; SQRT indicates that data were transformed and statistically analyzed with square roots.

As shown in Fig 2, compared with the FM diet, MBM diet and MBM+AA diet significantly increased muscle ATF4 total protein expression level by 4.0-fold and 2.2-fold, respectively, at 2 h after re-feeding *(P<0*.*05)*. The phosphorylation level of eIF2α were also higher in MBM and MBM+AA diet groups (1.9- and 1.7-fold) at 2h after re-feeding each diet in muscle *(P<0*.*05)*. Similarly, in liver, compared with the FM diet, MBM and MBM+AA diet significantly elevated the total expression level of ATF4 and phosphorylation level of eIF2α at 8 h after re-feeding *(P<0*.*05)*.

### IGF signaling pathway

**Fig 3:**
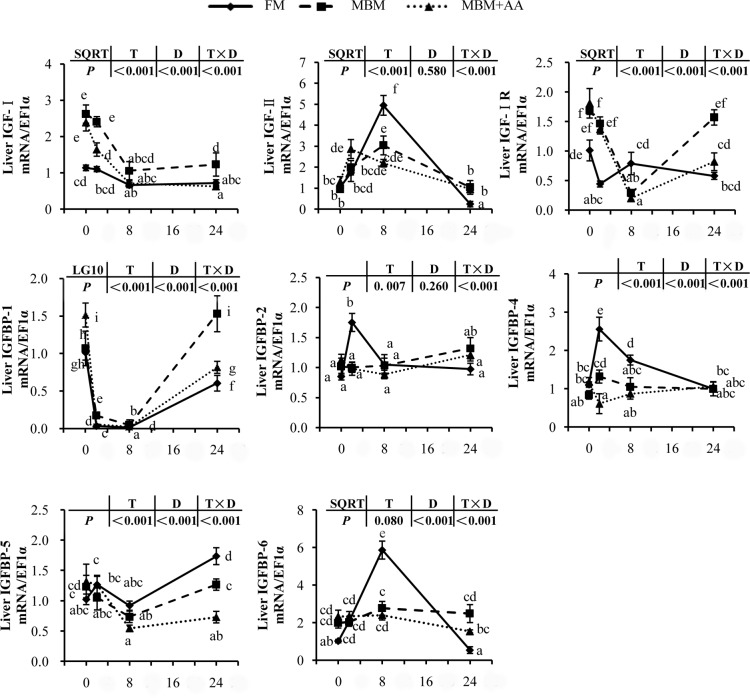
Post-prandial genes expression of IGF signaling pathway in the liver of juvenile turbot. Values are means ± SE, n = 6. All treatments were normalized to the expression level of 0 h FM diet group and by the reference gene EF1α. “a,b,c,d,e,f,g,h,i” were used to show significant difference between time points and diets (*P<0*.*05*). LG10 indicates that data were transformed and statistically analyzed with log transforms; SQRT indicates that data were transformed and statistically analyzed with square roots.

To assess the effect of different diet protein on the body endocrine, we measured the gene expression level of IGF-I, IGF-II, IGF-IR and IGFBPs (Fig 3). Compared with fasted, all the three groups of fish re-feeding with each diet significantly elevated the gene expression level of IGF-II, IGFBP-2, IGFBP-4, IGFBP-5 and IGFBP-6,and suppressed the mRNA expression level of IGF-I, IGF-IR and IGFBP-1 *(P<0*.*05)*. MBM diet significantly increased postprandial IGF-I expression level and decreased IGF-II expression level compared to those of the FM diet *(P<0*.*05)*. The gene expression level of IGF-IR was elevated in both MBM diet and MBM+AA diet compared to those of the FM diet group *(P<0*.*05)*. The IGFBPs presented a multitudinous regulation. MBM diet and MBM+AA diet significantly decreased the peak level of IGFBP-2, IGFBP-4, IGFBP-5 and IGFBP-6, but increased the level of IGFBP-1 than those of the FM diet *(P<0*.*05)*.

### Protein-degradation signaling pathway

**Fig 4:**
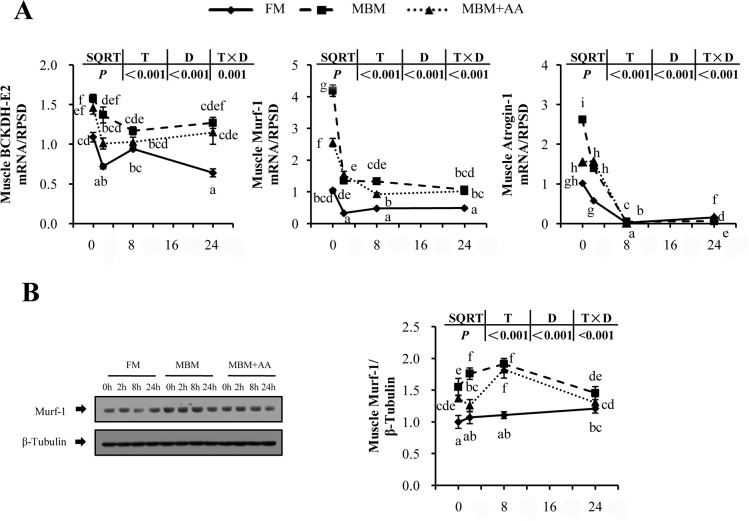
**Post-prandial mRNA (A) and total protein expression (B) of protein degradation in muscle of juvenile turbot.** Total protein of Murf-1 expression and the factors involved in ubiquination mRNA expression level (B) Values are means ± SENs, n = 6. All treatments were normalized to the expression level of 0 hrs FM and by the reference gene EF1α. “a,b,c,d,e,f,g,h,i”, “A,B,C” and “X,Y,Z” were used to show significant difference between time points and diets (*P<0*.*05*).

To investigate the underlying molecular mechanisms of decreased body protein deposition by MBM and MBM+AA diets, the muscle ubiquitination and branched-chain amino acids catabolism were analyzed in this study (Fig 4). The transcriptional level of Murf-1, Atrogin-1 and BCKDH-E2 in muscle decreased significantly after re-feeding *(P<0*.*05)*. Compared with the FM diet, MBM diet significantly increased the gene expression level of Murf-1, Atrogin-1 and BCKDH-E2 by about 3.0-fold, 1.6-fold and 0.5-fold at 0h after re-feeding, respectively *(P<0*.*05)*. Fish fed with MBM+AA diet also had significantly higher expression level of these genes than the fish fed with FM diet *(P<0*.*05)*. Furthermore, the total protein expression level of Murf-1 was also higher in MBM and MBM+AA diets than the FM diet group respectively *(P<0*.*05)*.

### Lipid, glucose and energy metabolism

**Fig 5:**
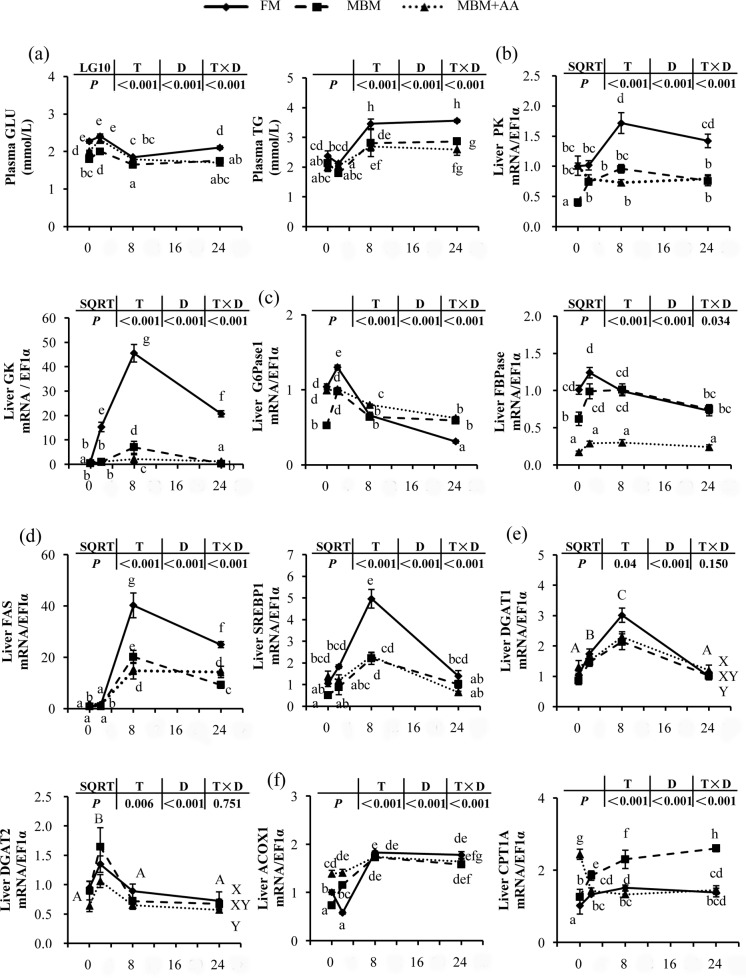
Post-prandial genes expression of intermediary metabolism in liver of juvenile turbot. The key enzymes involved in the liver glucose and lipid metabolism values are means ± SE, n = 6. The housekeeping gene EF1α was regarded as an internal control. ‘a,b,c,d,e,f,g,h’. ‘A,B,C,D’ and ‘X,Y,Z’ mean values were significantly different (*P<0*.*05*).

The plasma glucose (GLU) and triacylglycerol (TG) level and gene expression level of key enzymes involved in metabolism were characterized (Fig 5). Re-feeding induced higher plasma GLU and TG concentrations and reached their peaks at 2 h, 8h after re-feeding, respectively *(P<0*.*05)*. Compared with fish fed control group diet, fish fed MBM diets possessed lower concentrations of plasma GLU and TG *(P<0*.*05)*.

Compared with fasted, re-feeding significantly up-regulated the gene expression level of key enzymes involved in glycolysis (GK and PK), fatty acid synthesis (FAS and SREBP-1), triacylglycerol synthesis (DGAT1 and DGAT2) and gluconeogenesis (G6Pase1,2 and FBPase), while decreased the expression level of those associated with fatty acid oxidation (ACOX1 and CPT-1a) *(P<0*.*05)*.The mRNA expressions of key enzymes related to glycolysis for GK and PK and gluconeogenesis for G6Pase1 were lower in the MBM diet and MBM+AA diet group than the FM diet group *(P<0*.*05)*. The key enzymes involved in fatty acid synthesis and triacylglycerol synthesis, SREBP1, FAS, DGAT1 and DGAT2 were also down-regulated in the MBM diet and MBM+AA diet group compared with the FM diet group *(P<0*.*05)*. Conversely, CPT-1a, a fatty acid oxidation factor, showed highest mRNA expression level in MBM-fed group than those of the FM-fed group *(P<0*.*05)*.

## Discussion

### MBM diet reduced fish growth

Results of the present study demonstrated that replacement of 45% FM protein with MBM significantly decreased the feed efficiency, final body weight, specific growth rate and nutrient retention, which were consistent with previous studies conducted in white shrimp [28], gibel carp [29] and Japanese flounder [30]. Body protein and lipid synthesis contribute significantly to fish weight gain [31, 32]. The lower level of whole body protein and fat contents in fish fed with MBM diet indicated the poor growth performance of fish. Studies on longfin yellowtail and turbot showed the same decreased pattern of body protein and fat contents after FM replaced by other protein sources [23, 33]. Our data also showed that MBM+AA diet had no effect on improvement of the growth performance and nutrient contents. Previous studies demonstrated that compare with protein-bound AAs, crystalline AAs are more easily transported into plasma. Asynchronous absorption kinetics rendered the crystalline AAs used ineffectively [34]. To explore the underlying molecular mechanisms of poor growth performance after FM replaced by other protein sources, we focused on the responses of TOR, AAR and IGF signaling pathways and the expression of nutrient metabolism-related target genes and proteins in turbot.

### MBM diet inhibited postprandial TOR signaling pathway and activated AAR signaling pathway

Two distinct but complementary pathways TOR and AAR signal transduction pathways are responsible for sensing nutrient concentrations in cellular level [35–37]. Well-balanced diet is able to activate TOR signaling pathway and accelerate the initiation of mRNA translation to promote protein synthesis [38, 39]. The present study showed that compared with FM diet, MBM diet inhibited postprandial TOR signaling pathway with significantly decreased phosphorylation level of TOR, AKT, 4E-BP1 and S6. In rainbow trout, TOR signaling pathway was poorly activated by re-feeding of the lower protein-to-carbohydrates diet [40]. Seiliez *et al*. [24] also reported a similar work on rainbow trout that was consistent with our results. In contrast, amino acid deficiency activates GCN2 kinase and resulted in the inhibition of whole protein translation but increase the expression level of ATF4, a key regulator involved in AAR pathway that modulates a large number of genes (ATF3, CHOP, ASNS, REDD1) for the adaptation to dietary stress [41–43]. Our results also demonstrated that AAR signaling pathway were significantly up-regulated after fish fed MBM diet for one month. Previous study on MEF cells showed that cells cultivated in leucine limitation medium up-regulated the AAR and down-regulated mTORC1 pathway [44]. All of these results suggested that hypo-activated TOR pathway and hyper-activated AAR pathway decreased protein accretion in turbots fed MBM diet. That may give explanations for the lower whole body protein content and protein retention after fish fed with MBM and MBM+AA diets.

### MBM diet down-regulated the IGF signaling pathway

IGF system is a major hormone axis regulating intracellular protein synthesis and body metabolism [45, 46]. Nutritional status is also regarded as the key regulator of IGF system in fish [47, 48]. Previous study suggested that IGF pathway regulated somatic growth in vertebrates through integration of the IGF-I, IGF-II, IGF-IR and IGF-binding proteins (IGFBPs) [49]. Our research showed that MBM diet reduced the expression of IGF-II, whereas increased the IGF-I expression. IGF-IR expression was increased in the group fed MBM diet compared with the group fed FM diet. IGFBP-1 was known to be a negative regulator of IGFs activity [50]. Previous studies in zebrafish also presented that prolonged fasting increased the mRNA expression of IGFBP-1; however, re-feeding made a mark reduction in transcript of IGFBP-1 [50, 51], our study obtained similar results. MBM diet significantly elevated the expression of IGFBP-1 compared to FM diet group. On the contrary, IGFBP-2, IGFBP-4, IGFBP-5 and IGFBP-6 were shown as the positive actors in regulating IGFs [50, 52], while MBM diet down-regulated the expression of these interacting proteins. All these results demonstrated that MBM diet reduced the activities of the IGFs signaling pathway.

### MBM diet up-regulated protein-degradation signaling pathway

In mammals, muscle protein serves as the primary reservoir of amino acids that could be mobilized during fasting to provide a source of amino acids for energy production [53]. Loss of muscle protein occurs primarily through enhanced protein breakdown by activating the ubiquitin (Ub)-proteasome pathway [54, 55].The PI3K/AKT/FOXO pathway is necessary for ubiquitination, and this pathway is regulated by IGF system [13]. The Murf-1 and Atrogin-1play a critical role in the initiation of protein ubiquitination and are induced by nutrient deprivation [11]. Consistently, we observed that re-feeding reduced mRNA expression of Atrogin-1 and Murf-1 in this study. We also observed that MBM diet increased the level of Murf-1 and Atrogin-1, which were consistent with studies in Fine Flounder [14]. BCKDH-E2 is considered the key irreversible regulatory enzyme involved in branch-chain amino acid (BCAA) catabolism [15]. The BCKDH-E2 expression level was also higher in the MBM diet and MBM+AA diet than FM diet. The up-regulated BCAA catabolism would decrease the muscle concentration level of BCAA and further accelerated the muscle protein degradation process. In the present study, MBM diets increased gene expression of protein degradation were associated with the inhibition of IGF signaling pathway which is consistent with previous reports demonstrating that depressed IGF system could decrease the activity of PI3K, which stimulated protein degradation process [13, 56]. All the results presented a underlying molecular mechanism of regulation about lower muscle weight after turbots fed MBM and MBM+AA diet compared with turbots fed FM diet.

### MBM diet affected glucose, lipid and energy metabolism

As well as protein metabolism, glucose, lipid and energy metabolism were also affected by the diet protein sources. TOR and AAR signaling pathways not only control protein synthesis but also regulate the activities of the key enzymes in glucose, lipid and energy metabolism processes [6, 57]. Previous reports have demonstrated that the expressions of key enzymes involved in glucose, lipid and energy metabolism were regulated by the macronutrient composition of the diet in rainbow trout [24, 58, 59]. Our study showed that turbot fed MBM diet decreased the plasma concentrations of GLU and TG. Moreover, compared with the control group, the genes expression level of glycolysis (GK, PK) and lipogenesis (SREBP-1, FAS and DGAT1) were significantly decreased in MBM diet group, whereas the gene expression of CPT1A, a key regulator involved in fatty acid β-oxidation was significantly increased in MBM diet group. The MBM diets reduced TOR signaling activities and stimulated AAR activities, and inhibited the expression of genes involved in glucose, lipid anabolism, and elevated the gene expression level of fatty acid β-oxidation. Our results were consistent with the latest study showing that higher phosphorylation level of 4E-BP1 activated by TOR signaling pathway could elevate the level of glucose transport and glycolysis by increasing the level of GK and PK [60]. In addition, Li’s [61]study showed that TOR signaling pathway activated the expression of the positive regulator of fatty acid biosynthesis SREBP1by up-regulated the phosphorylation of S6K1.The SREBP1 is considered as a link regulator between TOR signaling pathway and fatty acid biosynthesis [62]. Study on rat demonstrated that CPT1 acted as a rate-limiting enzyme plays a pivotal role in regulating fatty acid β-oxidation [63]. The CPT1A is also a key regulator involved in energy metabolism [64]. Our study showed that turbot fed MBM diet decreased the biosynthesis of glucose and lipid and increased fatty acid β-oxidation. In all, these results demonstrated that FM replaced by MBM diet decreased the nutrient anabolism and increased catabolism which may give a well explain for the lower nutrient contents after fish fed MBM and MBM+AA diets.

## Conclusions

In summary, partial replacement of FM by MBM inhibited postprandial TOR and IGF signaling pathways and stimulated AAR signaling pathway. Those further induced higher level of protein degradation and glucose, lipid catabolism and lower level of nutrient anabolism. Similar molecular responses were observed after FM replacement by soybean meal in turbot [21]. These results suggested that it might represent a common reaction after FM substitution by other protein sources with reduced quality. These findings can be a good guide to improve the utilization of non-fishmeal protein sources in aquaculture.

## Supporting Information

S1 File The partial sequences of target genes in turbot (*Scophthalmus maximus*. L). A, IGFBP-4; B, IGFBP-5; C, IGFBP-6; D, Murf-1; E, Atrogin-1; F, BCKDH-E2; G, SREBP1. All the partial sequences of target genes in turbot were obtained through a degenerate PCR strategy in this study.
(PDF)
