# Supplementary material for: Comparative Study on the Cellular and Systemic Nutrient Sensing and Intermediary Metabolism after Partial Replacement of Fishmeal by Meat and Bone Meal in the Diet of Turbot (Scophthalmus maximus L.)
Source: PLoS One. 2016 Nov 1;11(11):e0165708. doi: 10.1371/journal.pone.0165708 (PMC5089717; doi:10.1371/journal.pone.0165708)
Supplement: S1 File — A, IGFBP-4; B, IGFBP-5; C, IGFBP-6; D, Murf-1; E, Atrogin-1; F, BCKDH-E2; G, SREBP1. All the partial sequences of target genes in turbot were obtained through a degenerate PCR strategy in this study. (PDF) [file pone.0165708.s001.pdf]

S1 File. The partial sequences of target genes in turbot (*Scophthalmus maximus.* L). A, IGFBP-4; B, IGFBP-5; C, IGFBP-6; D, Murf-1; E, Atrogin-1; F, BCKDH-E2; G, SREBP1. All the partial sequences of target genes in turbot were obtained through a degenerate PCR strategy in this study.

A. Scophthalmus maximus insulin-like growth factor binding protein-4 (IGFBP-4) mRNA, partial cds

5’-ATTAGCTGGTGGCAGTCTAGATCCCCTCGCTGCTCCAATGGCCCCGGCA
GCCTCAAGCCCGTCTTCTGATCCACACACCAGCACTTTCCCCGCTGACCGT
CACGCGCAGGGTGACACTGTTTAGCGTGGAAGTCTCCGTTCTTGTCACAGT
TTGGTATAGGGATCGTGAAGAGATCATCGTGTGTGCGGGTATTTGATGCCAG
TCTGTCCAGTGCTCTTTGTAGCTCAGCACGACATGGAGCCAGCGCCGCCTT
GGTCTCCTCCCTGGCTGTGTTGCTTCTCTGATTCGTGGATTTGGGAGGATGC
CGGGCCAGGGTCTTCTGTATGCAGCGCTTGTCTTGTGGACTGCACCGGATA
TTGCTGTTATTGGGGTGCTCGGG-3’

B. Scophthalmus maximus insulin-like growth factor binding protein-5 (IGFBP-5) mRNA, partial cds

5’-TGCACCAACGAGAAAGGATACAAGCCCGCTCACCCGCCCATAGATCGAG
ACTCTCGAGAGCAAGAGGACACCATCACCACAGAGATCACAGAGGAGTTC
CAGCCAGCCAAAGTGCCACTCCTTCCTAAAGACATTGTGAACAGTAAAAA
AGTCCATGCACTGCGCAAGGAGCAAAAGAGGAAGCAGGGCAAGCAGCGC
TTCATCGGCTCTCCCATGGACTACTCCCCTCTCCCCATCGACAAGCATGAGC
CAGAATTTGGTCCATGCAGAAGAAAATTGGATGGCATCATTCAGGGAATGA
AGGACACTTCTCGCGTAATGGCTCTCTCTTTGTACCTCCCAAACTGTGACC
G-3’

C. Scophthalmus maximus insulin-like growth factor binding protein-6 (IGFBP-6) mRNA, partial cds

5’-TACAGCCCCCTCCAGGCTCTGTTGCAGGGACGGGGCATTTGCGCCAAGC
ACAGCAGGACTGGTCCCACTGAGAGGCCCCCCCCACAGGTCCACATCCC
TCACACGGTGGTGACATTGAAAAAGCACCCTGCCGCAAGCTGCTCAATAG
TGTCCTGAGGGGTCTCGAGCTGACAATCTTCCAGTCTGACCGCGACATCTA
TATACCCAACTGTGACACTCGTGGCTTCTACAGGAAAAAG-3’

D. Scophthalmus maximus muscle ring-finger protein-1 (Murf-1) mRNA, partial cds

5'-CCTGCCCTGCCAGCACAACCTGTGTCGCGGCTGCGCCAACGACCTCTAC
GAGTCCAAGGACCCGTACCACTACTCCGGAGGAACCTTCCGCTGCCCCAC
CTGCCGCTTCGAGGTGATGCTGGACCGCCACGGCGTGTTCGGGCTGCAGA
GGAACCTGCTGGTGGAGAACATCATCGACATGTACAAGCAGCAGCAGGAG
AGTCGGGGCGGCGGCGAGGACCCGCCCCTGAAGGACAAAGACGCCAAGG
AGCCCAAGTGTAAGGAGCACGAAGACGAACGCATCAACATCTACTGCGTC
AGCTGCCAGACGCCAACCTGCTCCATGTGCAAGGTGTTCGGGCAGCACCA
GGACTGCGAGGTGTCGCCGCTGCTTGCCGTCTACCAGAGCCAGAAGAGCG
AGCTGTGCGCCGCCGTGGAGCAGCTGGCCGCAGGAAACGGCTGCGTCCAG
GCCGCCGTGGCCCAGATGGACGACACCTGCAAGGTGCTCCGGGACAACGG
GGAGCTGCAGAGGAGGCGTCTGGGCGAGAGCTTCGACCTGCTCTACGCCA
CCATGGACG-3'

E. Scophthalmus maximus Atrogin-1 mRNA, partial cds

5'-TCTCCGGGTCAGAGCTGGGTCAAGACCCAAGAGGGATGGAAAAAGACA
ACGGCCAACGACTCGAACAACAACGTCTCCGTGGAGAGCTTCTGCAAGGC
CGAGCAGGAATGTTTCAACAAGGAGAACTTGCTGCTGTCGCTCAGCTACG
ACATGGCTGCCAAGAAGAGGAAGAAAGACCTCATGAACAACAACGCCAA
GGTCCCCTATTTCCACAGGGAAAAGTGGATTTATGTTCATAAAGGAAGCAC
CAAGGAGCGCCACGGATATTGTACACTCGGAGAGGCCTTCAACCGCTTGGA
TCTCTGCAGCGCCATCAAGGATACGAGGAGATTTAATTACATCGTCAGACTT
CTGGAGCTTATCGCCAAGTCCCAGCTCCCCTCGCTCAGCGGAGTGGCGCAG
AAGAATTACATGAATATTCTGGAGAGAGTGGTACAGAAAGTCCTCGACGAC
CAGCAGAACGTCCGTCCGATCAAAGAGCTGCTGCAGACGCTCTACCTCTC
GCTCTGTGGCCTGGTTCAGGACATGGGCAAGTCCGTCCTGGTGGGGAACA
TCAACACCTGGCTGCGCCGCATGGAAAACATCCTGC-3'

F. Scophthalmus maximus branched-chain alpha-keto acid dehydrogenase E2 (BCKDH-E2) mRNA, partial cds

5'-GATGACGGCGGCGCTGAAGATTCCTCACTTCGGTTACTGCGACGAGGTC
GACCTCAGTCGCCTCGTCGCTCTGAGAGCTGAACTCAGATCTGTGGCTGAA
GGTCGAGGGGTCAAACTGAGCTACATGCCGTTCTTCATCAAGGCTGCTTCC
CTCTGTCTCCTCCACTTCCCCATTCTGAATGCTTCAGTGGACGACAGCTGCC
AGAACATCACCTACAAGGCGTCTCACAACATCGGGCTGGCGATGGACACC
ATTCAGGGTCTGCTGGTTCCCAACGTGAAGAACGTGCAGCTGCTCAGCGT
GTTGGATGTCGCTCTGGAGCTGAACCGTCTGCAGGCGCTGGGGTCAGCTG
GTCAACTGGGAACCAGCCACCTGAGCGGAGGAACCTTCACCTTGTCCAAC
ATCGGATCAATTGGGGGGACGTACGCCAAACCAGTGATTCTCCCTCCAGAG
GTCGCCATCGGAGCTCTGGGAAAAATCCAGGTCCTCCCTCGGTTCGACGGC
GGCGGTCAGGTGGTCCCGGCTCACATCATGAAGGTCAGCTGGTCGGCGGA

TCATCGCGT-3’

G. Scophthalmus maximus sterol regulatory element-binding protein 1 (SREBP1) mRNA, partial cds

5’-CATCGCCATCAGCGGAAAGCCCGTCGGCCAACCCCACAAAGGAGAAAA
GCGCACTGCCCACAACGCCATCGAAAAGCGCTATCGCTCCTCCATCAATGA
CAAGATCATTGAGCTCAAAGATCTGGTGGCTGGTACTGAGGCCAAGCTCAA
CAAGTCTGCAGTGCTGAGAAAAGCCATTGACTACATCCGTTACATACAGCA
GACTAACCAGAAACTCAAACAGGAGAACATGGCCCTGAAAATGGCCGCCC
AGAAAAACAAGTCTCTCAAAGACCTGGTAGCCATGGAAGTAGATGGACAG
GCTGATGTCAAGAACGAGCTGCCAACCCCCCCGGCTTCTGATGTGGGCTCT
CCGACGTCTTTCTCGCACTGTAGCAGTGACTCTGAGCCTGACAGTCCGATG
GGGGAAGACGCCAAGCCAAATGTGGGCGTGTTGGACACTTCGGCAGCAG
GCAGCAGTGCCGGCGGCATGTTGGACCGGTCCCGCATGGCGCTGTGTGCC
TTCACCTTCCTTTTCCTGTCTCTCAACCCTCTGGCTGCTTTGCTGTGTTCGT
CTGGCAGCAGCTCAGCTGGAAGCGCTGTAGCCTCCAGCGCCCATCATGCA
GGCAGGAGCGTTCTGGGTGTGGATATTGCAGCGGATTCGTGGGGCT-3’
